# Patient Safety in Surgery reviewer acknowledgement 2015

**DOI:** 10.1186/s13037-016-0099-4

**Published:** 2016-03-14

**Authors:** Philip F. Stahel, Pierre-Alain Clavien

**Affiliations:** Denver Health Medical Center, Denver, USA; University Hospital Zurich, Zurich, Switzerland

## Abstract

The editors of *Patient Safety in Surgery* would like to thank all of our reviewers who have contributed to the journal in Volume 9 (2015).

**Shay Bess**

United States of America

**Evalina Burger**

United States of America

**Nathan Butler**

United States of America

**Ted Clarke**

United States of America

**Michael Dayton**

United States of America

**Joan Figueras**

Spain

**Joerg Franke**

Germany

**Beat Gloor**

Switzerland

**Andrew Grose**

United States of America

**Dieter Hahnloser**

Switzerland

**Mark Hake**

United States of America

**Eric Mark Hammerberg**

United States of America

**Christoph-E. Heyde**

Germany

**Markus Huber-Lang**

Germany

**Kyros Ipaktchi**

United States of America

**Jeffry Kashuk**

United States of America

**Fernando Kim**

United States of America

**Philipp Lenzlinger**

Switzerland

**Cyril Mauffrey**

United States of America

**Wilson Molina**

United States of America

**Leslie Ness**

United States of America

**Giuseppe Nigri**

Italy

**Lena Nilsson**

Sweden

**Yohan Robinson**

Sweden

**Marius Scarlat**

France

**Beth Schulte**

United States of America

**David Seligson**

United States of America

**Todd Vanderheiden**

United States of America

**Michael Victoroff**

United States of America

**Sebastian Weckbach**

United States of America

**Heather Young**

United States of America

**George Youngson**

United Kingdom

**Navid Ziran**

United States of AMerica

